# A new bimodal approach for sentinel lymph node imaging in prostate cancer using a magnetic and fluorescent hybrid tracer

**DOI:** 10.1007/s00259-023-06522-8

**Published:** 2023-11-24

**Authors:** Bianca Michalik, Svenja Engels, Maximilian C. Otterbach, Jorina Frerichs, Paula E. Suhrhoff, Matthias N. van Oosterom, Martin H. Maurer, Friedhelm Wawroschek, Alexander Winter

**Affiliations:** 1 University Hospital for Urology, Klinikum Oldenburg, Department of Human Medicine, School of Medicine and Health Sciences, Carl Von Ossietzky University Oldenburg, Oldenburg, Germany; 2 University Institute for Diagnostic and Interventional Radiology, Klinikum Oldenburg, Department of Human Medicine, School of Medicine and Health Sciences, Carl Von Ossietzky University Oldenburg, Oldenburg, Germany; 3 Interventional Molecular Imaging Laboratory, Department of Radiology, Leiden University Medical Center, Leiden, The Netherlands

**Keywords:** Prostate cancer, Sentinel lymph node, Image-guided surgery, SPION, Indocyanine green

## Abstract

**Purpose:**

To obtain initial data on sentinel lymph node (SLN) visualisation by pre-operative magnetic resonance imaging (MRI) and intra-operative bimodal SLN identification using a new magnetic fluorescent hybrid tracer in prostate cancer (PCa) patients.

**Methods:**

Ten patients at > 5% risk for lymph node (LN) invasion were included. The day before surgery, a magnetic fluorescent hybrid tracer consisting of superparamagnetic iron oxide nanoparticles (SPION) and indocyanine green was transrectally injected into the prostate. Five hours after injection, transversal pelvic MRI scans were recorded and T2*-weighed images were screened for pelvic LNs with SPION uptake. Intra-operatively, magnetically active and/or fluorescent SLNs were detected by a handheld magnetometer and near-infrared fluorescence imaging (FI). Extended pelvic lymph node dissection (PLND) and radical prostatectomy completed the surgery. All resected specimens were checked ex situ for magnetic activity and fluorescence and were histopathologically examined.

**Results:**

Pre-operative MRI identified 145 pelvic LNs with SPION uptake. In total, 75 (median 6, range 3‒13) magnetically active SLNs were resected, including 14 SLNs not seen on MRI. FI identified 89 fluorescent LNs (median 8.5, range 4‒13) of which 15 LNs were not magnetically active. Concordance of the different techniques was 70% for pre-operative MRI vs. magnetometer-guided PLND and 88% for magnetic vs. fluorescent SLN detection.

**Conclusion:**

These are the first promising results of bimodal, magnetic fluorescent SLN detection in PCa patients. Our magnetic fluorescent hybrid approach provides the surgeon a pre-operative lymphatic roadmap by using MRI and intra-operative visual guidance through the application of a fluorescent lymphatic agent. The diagnostic accuracy of our new hybrid approach has to be evaluated in further studies.

**Trial registration:**

DRKS00032808. Registered 04 October 2023, retrospectively registered.

## Introduction

Through the rise of minimally invasive and/or robotic surgery in prostate cancer (PCa), image-guidance becomes particularly important [1]. Especially during robot-assisted radical prostatectomy, image-guidance can improve visualisation and selective dissection of pelvic lymph nodes directly draining from the prostate [2]. Excellent sentinel lymph node (SLN) detection rates [3] as well as oncologic outcome in terms of biochemical recurrence-free survival can be reached through application of a bimodal, radioactive and fluorescent hybrid tracer [4]. The hybrid tracer combines 99 m-Technetium (^99m^Tc)-nanocolloid and indocyanine green (ICG) to enable pre-operative lymphoscintigraphy through single photon emission computed tomography with low-dose computed tomography (SPECT/CT) as well as intra-operative guidance by a laparoscopic gamma-probe and near-infrared (NIR) fluorescence imaging [5, 6]. This bimodal approach, which combines the advantages of both techniques, thus provides the surgeon a lymphatic road map for careful surgical planning and excellent intra-operative guidance to prevent extensive lymph node dissection. Despite the hybrid tracer procedure is highly reliable for nodal staging in different malignancies [7], it may possibly be not easy to implement the procedure in clinical routine use everywhere due to logistic challenges. For example, the dependence on radioisotopes or nuclear medicine infrastructure and the associated legal requirements may impose restrictions on patient planning and require special hospital logistics. The hybrid procedure is therefore routinely applied by only a few medical centres to date [8]. Superparamagnetic iron oxide nanoparticles (SPION) have been introduced as radiation-free alternative to conventional radioactive agents for SLN marking in breast cancer [9]. The diagnostic accuracy of magnetic labelling of SLNs has subsequently been confirmed in PCa [10, 11]. Like a SPECT/CT lymphoscintigraphy of radioactive labelled SLNs, SPION uptake in SLNs can be pre-operatively visualised through magnetic resonance imaging (MRI) [12–14]. The next logical step was thus to combine SPION and ICG to obtain a magnetic fluorescent hybrid tracer to enable radiation-free, bimodal surgical SLN dissection suited for clinically routine use and which may facilitate minimally invasive pelvic lymph node dissection (PLND) in PCa patients. General applicability of this procedure has already been shown in animal model [15, 16] and first in-human surgeries [17]. With this study, we aimed to obtain initial data on pre-operative as well as intra-operative SLN identification after administration of the new magnetic fluorescent hybrid tracer in PCa patients. Results of pre-operative SLN visualisation by using MRI were compared with the results of intra-operative SLN detection by either mode and histopathological results were contemplated.

## Methods

### Patients

Ten PCa patients who were administered to the University Hospital for Urology, Klinikum Oldenburg, Germany between March and May 2023, and were scheduled to open retropubic radical prostatectomy combined with PLND were enrolled in this study. All patients were at an individual risk for lymph node invasion (LNI) of > 5% according to our nomogram [18]. After careful checking for possible contraindications to ICG administration, e.g., iodine allergy, thyroid hyperfunction, thyroidal adenoma, or kidney insufficiency, patients were offered additional SLN marking with ICG. Patients were informed orally and in writing about the procedure and possible associated risks. All patients gave written informed consent, and the Medical Ethics Committee of the Carl von Ossietzky University Oldenburg approved this retrospective study (No. 2023–145).

### Tracer injection

Tracer injection took place the day before radical prostatectomy (median 21.75 h, range 18.15 – 25.53 h before surgery). The magnetic fluorescent hybrid tracer was designed according to the radioactive fluorescent hybrid tracer [6] and adjusted during first pre-tests by our group [15, 17]. 100 µl of 5 mg/ml ICG solution (25 mg ICG powder; Verdye, Diagnostic Green, Germany; dissolved in 5 ml sterile water) were mixed with 2 ml SPION (Magtrace, Endomag, Cambridge, UK). The total tracer volume was transrectally injected under ultrasound guidance in three deposits into each prostatic lobe, respectively. The injection scheme was designed to preferably trace the lymphatic drainage of the whole organ [11]. 

### Pre-operative magnetic resonance imaging (MRI)

The day before surgery, approximately 5 h after tracer injection (median 5.25 h, range 1.92 – 6.00 h), MRI was performed to pre-operatively visualise pelvic lymph nodes with SPION uptake [14]. Transversal T1-, T2-, and T2*-weighted sequences were recorded at 1.5 Tesla (MAGNETOM Aera, Siemens Healthineers, Erlangen, Germany) [13]. Along a predefined anatomical template in the internal and external iliac, obturator fossa, periprostatic and paravesical regions of each side of the body, pelvic lymph nodes with reduced signal intensity on T2*-weighted images were counted. According to our previous studies any lymph node showing a drop in signal intensity due to SPION uptake was defined as SLN [13]. Images were appraised by a radiologist experienced in magnetic sentinel diagnostics using MRI and by a urologist surgeon in an interdisciplinary expert assessment.

### Surgical procedure and histopathological examination

**Fig. 1:**
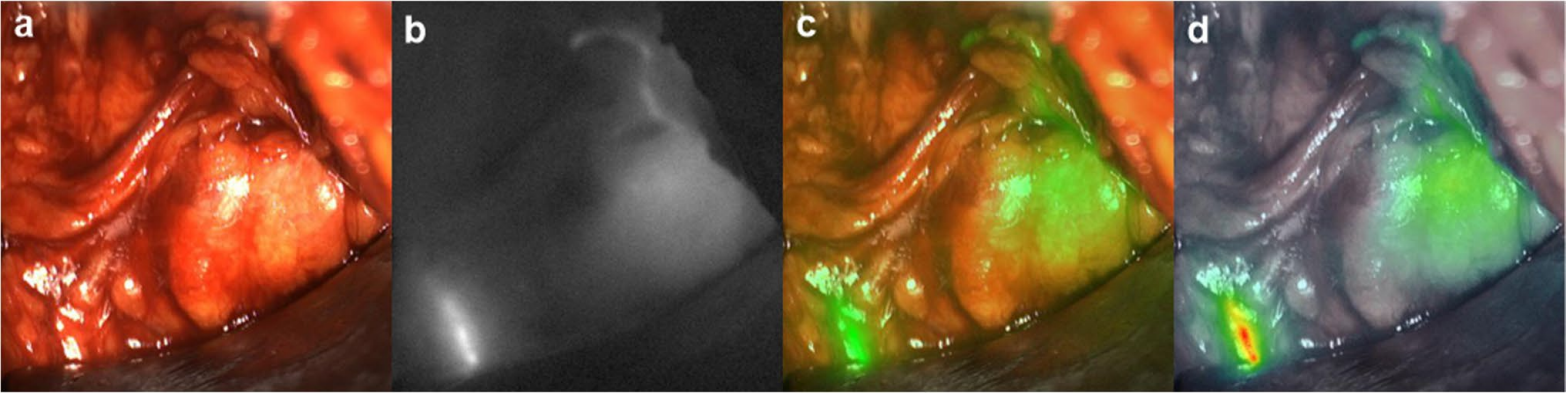
Example image series as provided from the near-infrared (NIR) optical imaging system with displays of white light (**a**), monochromatic NIR light (**b**), overlay mode (**c**), and intensity gradient (**d**) images of the fluorescence signal. In situ images show a fluorescence labelled lymph node as well as a lymphatic vessel (down left) in the right external iliac region

During surgery, the pelvic regions as described above were systematically searched for magnetic activity by a handheld magnetometer probe (Sentimag, Endomag, Cambridge, UK) [9] considering the pre-operative MRI findings and visually searched for fluorescence by a NIR optical imaging system (QUEST SPECTRUM 3, Olympus, Hamburg, Germany). To facilitate visual guidance as well as detection of the fluorescence signal, the NIR imaging system provides simultaneous, parallel displays of white light, monochromatic NIR light, overlay mode, and intensity gradient images of the fluorescence signal (Fig. 1). Each spot identified in situ by any mode, i.e., by magnetic activity and/or fluorescence, was surgically removed, separated from fatty tissue to individual lymph nodes and re-measured ex situ for magnetic activity as well as fluorescence. Concordance was defined as the percentage of resected lymph nodes visualised by both modalities or by neither modality. The surgery was completed by extended pelvic lymph node dissection (ePLND) and radical prostatectomy. Each resected specimen was formalin-fixed for approximately 24 h, routinely processed, cut into 2 – 8 mm transverse slices and embedded into paraffin. Sections of 4 – 5 µm were stained with haematoxylin–eosin and microscopically analysed for tumour infiltration by an experienced uropathologist.

## Results

Ten patients with PCa and an individual risk for LNI of > 5% according to our nomogram [18] were included in this analysis (Table 1). None of the patients exhibited any adverse reaction to tracer injection.

**Table 1:** Patient summary of clinical, therapeutic and post-operative parameters

Patient characteristics	Median (range)
Clinical parameters	
Age (years)	70.5 (58–83)
BMI (kg/m^2^)	27.5 (24–33)
PSA (ng/ml)	10.8 (2.4–22.5)
Percentage of positive biopsy cores	44 (25–70)
ISUP grade at biopsy*	2.5 (1–4)
1	2
2	3
3	3
4	2
Clinical tumour category*	-
1c	8
2b	1
2c	1
Pre-operative risk for LNI (%)**	21 (6–58)
Therapeutic parameters	
Spots with SPION uptake identified on pre-operative MRI (per patient)	14 (7–21)
Resected magnetically active lymph nodes (per patient)	6 (3–13)
Resected fluorescent lymph nodes (per patient)	8.5 (4–13)
Post-operative parameters	
ISUP grade*	2 (1–3)
1	1
2	7
3	2
Pathological tumour category*	-
2c	6
3a	3
3b	1
Tumour volume (ccm)	3.4 (1–6)
Pathological nodal status	0

* Data of individual categories are numbers. ** According to our nomogram [18]. *BMI* body mass index, *ISUP* International Society of Urological Pathology, *LNI* lymph node invasion, *MRI* magnetic resonance imaging, *PSA* prostate specific antigen, *SPION* superparamagnetic iron nanoparticles

### Pre-operative MRI and magnetometer-guided PLND

**Fig. 2:**
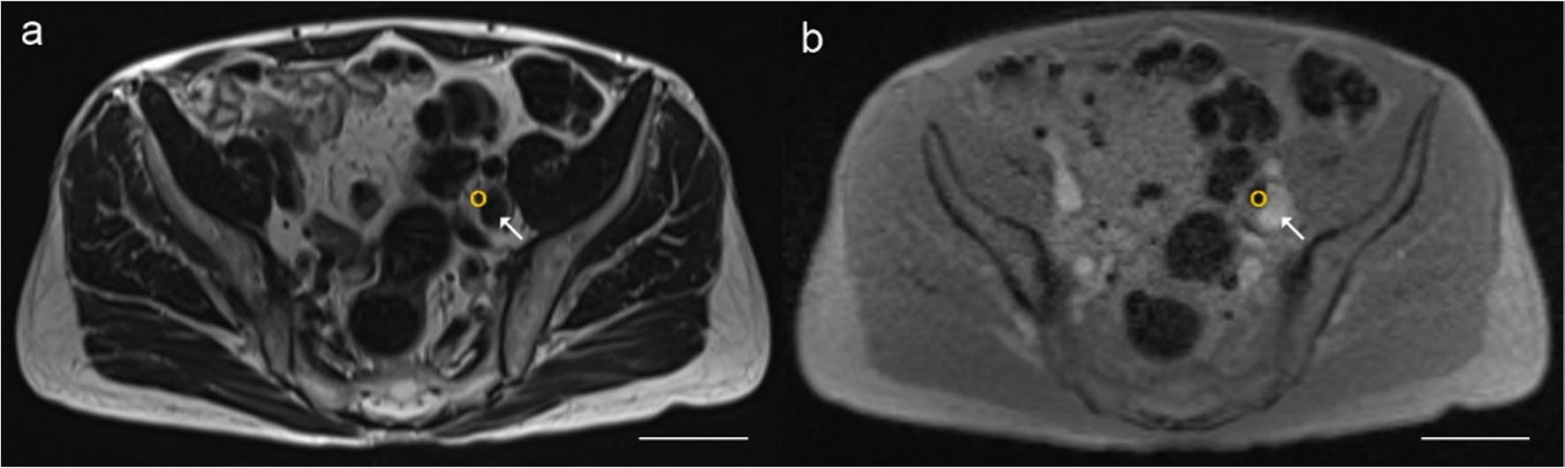
Pre-operative transversal magnetic resonance imaging scans of the pelvis with (**a**) T2-weighted and (**b**) T2*-weighted images showing lymph nodes with uptake of superparamagnetic iron nanoparticles in the left external iliac region (yellow circle). Arrow depicts the left external iliac vein. Up, ventral; down, dorsal; scale bar, 50 mm

Within our PLND template, pre-operative MRI identified in total 145 spots with reduced signal intensity on T2*-weighted images indicating lymph nodes with SPION uptake (Fig. 2). Magnetometer-guided PLND revealed in total 75 magnetically active SLNs, including 14 SLNs not seen on pre-operative MRI. As the evaluation of unmarked, i.e., negative lymph nodes and the direct matching of pre- and intra-operatively detected SLNs are rather limited, the calculation of a concordance rate between pre- and intra-operative SLN detection is not possible. We therefore calculated the concordance between the two techniques in detecting SLNs with SPION uptake in each of the individual anatomical regions within our PLND template. In 39 regions, magnetically active SLNs could be detected pre- as well as intra-operatively and in 31, no SLNs were detected. In 28 regions, SLNs were only visualised by pre-operative MRI and 2 only during magnetometer-guided PLND. The resulting concordance of magnetometer-guided PLND with respect to pre-operative MRI was 70% with a false negative rate of 28% and an additional diagnostic value, i.e., false positive rate, of 2%.

### NIR fluorescence imaging and magnetometer-guided PLND

**Fig. 3:**
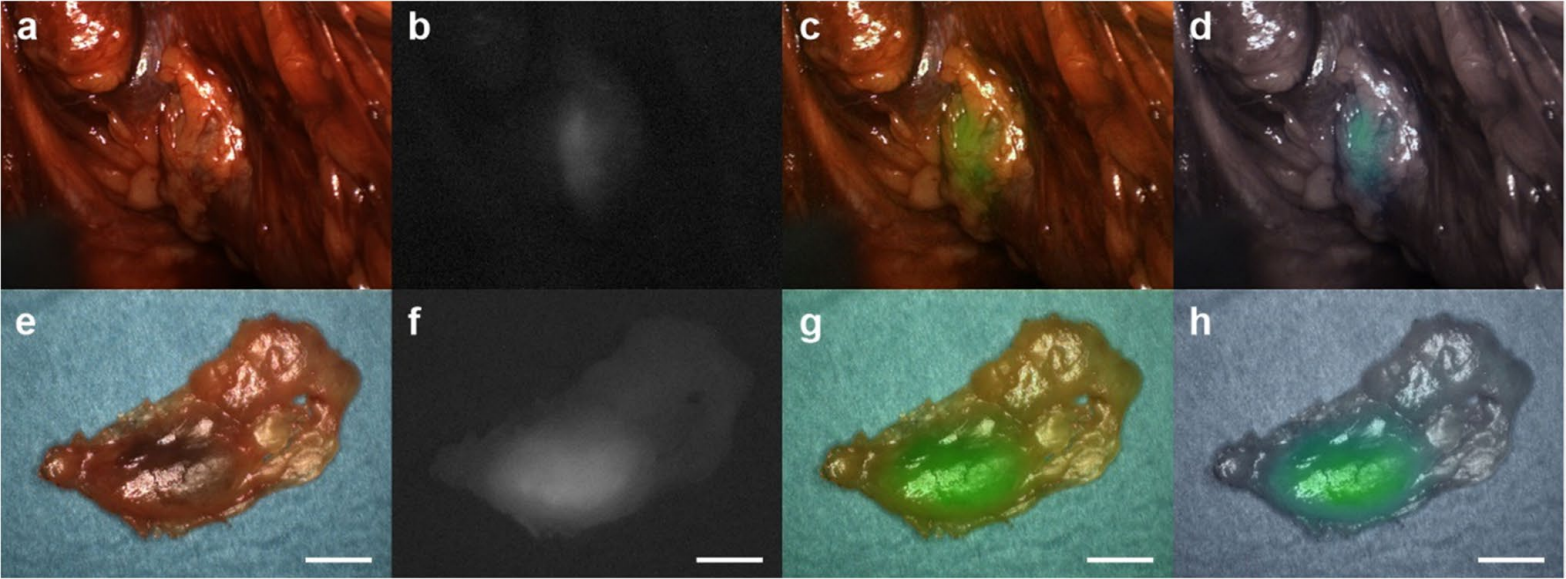
Images of a left side pelvic lymph node recorded by the near-infrared (NIR) imaging system with (**a**,**e**) white light, (**b**,**f**) monochromatic NIR, (**c**,**f**) overlay, and (**d**,**h**) intensity gradient of the fluorescence signal. **A**-**d** shows a fluorescent spot adjacent to the left external iliac vein. **E**–**h** shows the corresponding lymph node prepared ex situ. Scale bar 10 mm. Images are from the same patient as in Fig. 2

Intra-operative NIR fluorescence imaging revealed 35 fluorescent spots and magnetometer-guided PLND identified 42 magnetically active spots (Table 2). Eight spots detected in situ were magnetically active only and one was fluorescent only (Table 2). The resulting concordance between the two techniques was 88% (Table 2). In total, ex situ preparation of the resected specimens revealed 140 individual lymph nodes of which 75 were magnetically active and 89 were fluorescent, respectively (Table 2). All but one of the magnetically active lymph nodes were also fluorescent resulting in an overall concordance of 89% (Table 2). In one case, we dissected one paravesical SLN not visible in preoperative imaging which certainly would have been left behind during standard ePLND. An example of a fluorescent spot intra-operatively recorded in the external iliac region and the corresponding lymph node prepared ex situ is presented in Fig. 3. None of the resected lymph nodes contained metastases.

**Table 2:** Magnetically active (M +) and/or fluorescent (F +) spots detected in situ by a handheld magnetometer and/or NIR fluorescence imaging with corresponding numbers of lymph nodes prepared and re-measured ex situ

	M + /F +	M-/F-	M + /F-	M-/F +	total	concordance
In situ	34	34	8	1	77	88%
Ex situ	74	50	1	15	140	89%

Data are numbers if not specified otherwise

## Discussion

Through the application of a magnetic fluorescent hybrid tracer, we were able to combine pre-operative MRI and magnetometer-guided as well as NIR fluorescence imaging guided PLND in PCa patients. We could pre-operatively identify SLNs with SPION uptake on pelvic MRI scans as well as intra-operatively detect magnetic SLNs by a handheld magnetometer probe and fluorescing SLNs by NIR fluorescence imaging in all patients. Concordance of the different techniques was 70% for pre-operative MRI vs. intra-operative magnetometer-guided PLND and 88% for intra-operative magnetic vs. fluorescent SLN detection.

We could identify SLNs with SPION uptake on pre-operative pelvic MRI scans in all patients. As shown in previous studies on breast cancer [12], PCa [13, 14], and penile cancer [19], pre-operative SLN visualisation on MRI was thus generally feasible. In our study, a relatively high number of possible SLNs per patient has been pre-operatively identified on MRI scans which is in line with our previous studies using the SPION tracer alone with medium 17.5 (IQR 12‒22.5) SLNs pre-operatively visualised and 9 (6‒12) SLNs surgically removed [13]. Studies using a radioactive SLN tracer identified median 6 (IQR 3‒9) SLNs on pre-operative pelvic SPECT/CT lymphoscintigraphy and 4 (2‒6) SLNs were intra-operatively detected with a gamma probe and surgically removed [20]. Similar pre-operative visualisation and intra-operative detection rates could be observed using the radioactive fluorescent hybrid tracer with median 3 (IQR 2‒4.5) SLNs visualised and 2 (1‒4) SLNs surgically removed [3]. Due to the fundamentally different chemical and physical properties of the different imaging agents, however, a direct comparison has to be interpreted with caution. As discussed earlier, MRI might identify more SLNs due to its high spatial resolution and its high sensitivity to very small concentrations of SPION [13]. This could possibly lead to an overestimation of the number of pelvic SLNs with SPION uptake identified on pre-operative pelvic MRI scans. Due to the high number of lymph nodes mapped, it is challenging to match individual lymph nodes identified pre- and intra-operatively on a one-to-one basis. We therefore calculated the concordance between pre- and intra-operative SLN detection based on the individual anatomical regions within our PLND template, resulting in a concordance of 70%. Considering the small number of patients included in our study, this concordance rate has to be interpreted carefully. Despite these clear limitations, our results suggest that pre-operative SLN visualisation on MRI may provide the surgeon a lymphatic roadmap for PLND comparable to SPECT/CT lymphoscintigraphy using the conventional radioactive tracer.

During PLND, we could detect magnetically active as well as fluorescent SLNs in each patient. The number of resected magnetically active SLNs did not differ from that reported in our previous studies [10, 21] suggesting that the addition of ICG to SPION did not alter lymphatic uptake and distribution properties of the tracer. Among the resected lymph nodes, there were more fluorescent nodes identified than magnetically active SLNs. It is likely that the magnetic fluorescent hybrid tracer may also contain “free” ICG which has not bound to SPION due to the non-covalent binding reaction [15]. ICG alone acts as a lymphatic agent that, because of its small hydrodynamic diameter of 1.2 nm, has the potential to migrate beyond SLNs which may result in marking of secondary lymphatic landing sites [22]. In our study, only one specimen had been identified solely by NIR fluorescence during surgery. The higher amount of lymph nodes marked by ICG than by SPION became apparent only after ex situ preparation and re-measurement of all resected specimens. Our data thus suggest that intra-operative guidance of PLND by NIR fluorescence imaging may not necessarily lead to a more extensive surgical removal of lymph nodes considering a hybrid tracer approach.

As seen in applications of the radioactive fluorescent hybrid tracer, intra-operative NIR fluorescence imaging facilitated the identification of SLNs located in lymphatic fatty tissue [7]. A more favourable signal to background ratio might furthermore improve SLN detection during surgery. Especially in periprostatic regions where strong signal originating from the prostatic injection sites may mask SLN identification using the magnetic modality alone the hybrid approach could be advantageous due to a lower tissue penetration depth of the fluorescence signal. According to our previous studies using the SPION tracer, about 4% of the pre-operatively visualised SLNs on MRI were located periprostatically [13] whereas only about 0.3% of the surgically resected SLNs stemmed from periprostatic regions pooled over more than 800 patients who underwent magnetometer-guided PLND in our centre [10]. The spatial resolution of the magnetometer probe used in our studies is about 2 mm [9] which can be helpful in identifying SLNs located in deep tissue but can be misleading in areas with strong background signal. Considering the small number of patients included in this pilot study, the additional diagnostic value of applying a magnetic fluorescent hybrid approach cannot be evaluated and should be investigated in future studies with a larger patient cohort.

There is ongoing debate on the definition of SLN diagnostics in PCa [8]. In line with our previous studies using the SPION tracer [10, 11, 13, 21], we applied a wider SLN definition than is generally used in other studies or other tumor entities using the conventional radioactive or the radioactive fluorescent hybrid tracer [3, 20]. Due to the rather complex and highly variable lymphatic drainage pattern in PCa when compared to other tumour entities, our protocol is designed to perform a lymphatic mapping of the whole prostate and to surgically resect any magnetically active node [10, 11, 13, 21]. A retrospective analysis of our data on magnetometer-guided PLND in intermediate and high risk PCa patients revealed that the magnetic activity of the resected lymph nodes did not correlate with lymph node involvement [21]. Instead, about 17% of node positive patients would have been missed when applying the 10% rule based on node magnetic activity level [21]. In this pilot study, however, no lymph node metastases could be detected and conclusions on the diagnostic accuracy of our hybrid approach cannot be drawn.

If the reliability of intra-operative NIR fluorescence guidance in combination with pre-operative MRI can be confirmed in future studies with larger sample sizes, the application of the magnetic fluorescent hybrid tracer for SLN visualisation could be especially useful in minimally invasive, robot assisted surgeries. To our knowledge, there are no magnetometer probes for laparoscopic use commercially available, yet, but only in pre-clinical testing [16, 23, 24]. The magnetic fluorescent hybrid tracer could thus be a radiation-free alternative to the radioactive fluorescent hybrid tracer for bimodal SLN mapping in PCa.

## Conclusion

Our data present the first promising results of a radiation-free, bimodal SLN visualisation in PCa patients. Our magnetic fluorescent hybrid tracer approach provides the surgeon a pre-operative lymphatic roadmap by using MRI and intra-operative visual guidance through the application of a fluorescent lymphatic agent. The diagnostic accuracy of our new hybrid approach has to be evaluated in a future, prospective study with a larger, carefully selected patient cohort.

## Data Availability

The dataset analysed during this study are available from the corresponding author on reasonable request.
